# Conservation Investment for Rare Plants in Urban Environments

**DOI:** 10.1371/journal.pone.0083809

**Published:** 2013-12-31

**Authors:** Mark W. Schwartz, Lacy M. Smith, Zachary L. Steel

**Affiliations:** 1 John Muir Institute of the Environment, University of California Davis, Davis, California, United States of America; 2 Department of Environmental Science & Policy, University of California Davis, Davis, California, United States of America; 3 Graduate Group in Ecology, University of California Davis, Davis, California, United States of America; University of Kent, United Kingdom

## Abstract

Budgets for species conservation limit actions. Expending resources in areas of high human density is costly and generally considered less likely to succeed. Yet, coastal California contains both a large fraction of narrowly endemic at-risk plant species as well as the state's three largest metropolitan regions. Hence understanding the capacity to protect species along the highly urbanized coast is a conservation priority. We examine at-risk plant populations along California's coastline from San Diego to north of San Francisco to better understand whether there is a relationship between human population density and: i) performance of at-risk plant populations; and ii) conservation spending. Answering these questions can help focus appropriate strategic conservation investment. Rare plant performance was measured using the annualized growth rate estimate between census periods using the California Natural Diversity Database. Human density was estimated using Census Bureau statistics from the year 2000. We found strong evidence for a lack of a relationship between human population density and plant population performance in California's coastal counties. Analyzing US Endangered Species expenditure reports, we found large differences in expenditures among counties, with plants in San Diego County receiving much higher expenditures than other locations. We found a slight positive relationship between expenditures on behalf of endangered species and human density. Together these data support the argument that conservation efforts by protecting habitats within urban environments are not less likely to be successful than in rural areas. Expenditures on behalf of federally listed endangered and threatened plants do not appear to be related to proximity to human populations. Given the evidence of sufficient performance in urban environments, along with a high potential to leverage public support for nature in urban environments, expenditures in these areas appear to be an appropriate use of conservation funds.

## Introduction

Determining the degree to which limited resources should be applied to conservation in urban environments remains a critical challenge [1]–[3]. This challenge is acute in California where a large number of at-risk plant species are restricted to regions of high human density [4]–[6]. Limited conservation resources require strategic investment in conservation (e.g., [7]). The land cost associated with urban environments is typically far higher than rural areas [8]. Further, conservation opportunities within an urbanized landscape often consist of small, isolated fragments [9]–[11]. Small, isolated fragments of natural habitats may have lower species richness than larger natural areas [12], and populations within these sites may be at an elevated risk of extinction [13]–[16]. Rare species, particularly plants and insects, are often spatially associated with urban environments [8], [15], [17]. Further, the social value of conserving biodiversity that is accessible to urban populations may be high [18]–[22]. Thus, Lawson et al. [23] raised considerable interest in demonstrating that extant populations of plants in urban areas do not seem to have different population growth rates than those in rural environments.

A constraint of the Lawson et al. [23] study is that it did not distinguish performance of species among habitat types. We might predict that particular types of habitats (e.g., serpentine outcrops) may be relatively resilient to the kinds of changes in urban environments that put rare plant species at risk. For example, we might expect that the threat of invasive plant species to native populations may be higher in wetland habitats than in edaphically stressful environments (e.g., serpentine). Alternatively, other habitat types (e.g., agricultural landscapes with low human density) may also be vulnerable to similar degradation as in urbanized environments [15]. Protected wetlands, for example, may be threatened by invasive species, nutrient additions, environmental toxins, and other impacts across gradients of human density.

We have two distinct objectives in this paper. First, we further test the hypothesis of Lawson et al. [23] —that urbanization has no detectable effect on performance across a rural to urban gradient. We expand the evaluation of this hypothesis by taking a much closer look at potential confounding factors that may mask a relationship, including asking whether habitat types express differential performance relationships across the rural to urban gradient. We predict that species in some urban habitat types may be more resilient to urbanization than others. If so, then focusing on habitat types that are resilient to urbanization can help increase effectiveness of urban conservation efforts. To assess this hypothesis, we replicate the methods of Lawson et al. [23] to examine population performance of plant species across a human population density gradient. Specifically, we used the California Natural Heritage plant observation data (California Natural Diversity Database, CNDDB [24]) to determine population trends where repeated population size data are available. We then classified species into different habitat types and life forms to examine performance as a function of human density (people/km^2^) in more detail.

Our second objective is to assess the degree to which conservation investment in California may be biased by human density. One argument is that the high cost of urban conservation places too high a demand on limited funds relative to the benefits obtained [16]. The results of Lawson et al. [23] challenge the notion that investment in urban conservation is wasted investment. We cannot, unfortunately, relate government spending directly to plant population outcomes because our plant performance data are neither temporally nor spatially linked to expenditures. We can, however, assess patterns in spending on behalf of federally listed endangered plants to determine if it appears inappropriate, given plant performance. We hope that comparing and contrasting these two independent data sets help us to develop better strategies for how expenditures can be effectively applied to plant conservation in California, where there is a high fraction of urban-associated rare species and many of the resources for conservation are generated [25]. Together, these two pieces of information can help guide appropriate investment in conserving plant diversity within the urbanized and urbanizing California landscape.

## Methods

**Figure 1:**
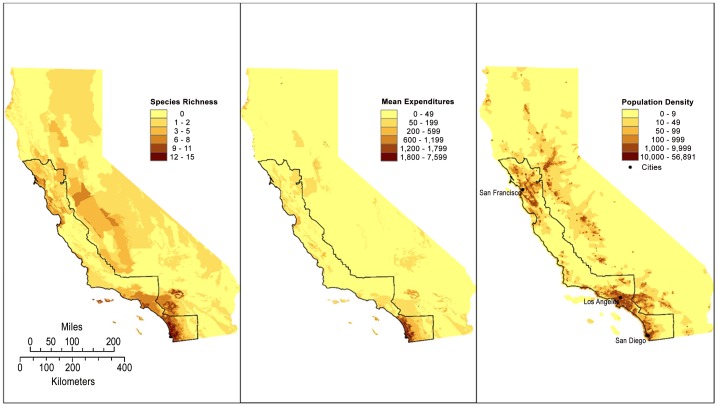
Maps of California highlighting population and expenditure attributes. The outline represents the study area for examining λ_ann_ relative to human density. Color shades represent: (A) species richness of federally listed plant species in California (number of species per square kilometer); and (B) mean annual expenditures for federally listed plant species (dollars per square kilometer per year). Locations of major California cities are included for reference on map (C).

We defined the study area to encompass the richest region of rare species in the state of California [6], focusing on at-risk plant species along coastal California from the Mexican border through the San Francisco Bay Area. Our study area was composed of the 17 counties that border the coastline, including San Francisco Bay, from San Diego to Sonoma County (Fig. 1a). Within this region, we aggregated all plant population census data available through the California Natural Diversity Database (CNDDB) [24]. The CNDDB is a spatially explicit database of rare plant and animal occurrences within California. Tracked plants are those defined by the California Native Plant Society [26] as at-risk. Plant occurrence records are based on sightings by both professional and amateur botanists. All records are then vetted by professional biologists before being entered into the database [24]. We use data on at-risk plant populations that contained species, location, and dates of at least two quantitative estimates of population size across a time interval of one year or more. Most CNDDB population locations do not contain repeated estimates of population size, a requirement in order to estimate performance.

We aggregated data on 1,682 population change estimates among 253 species and subspecies across 795 locations. Data included observations from 1897 through 2007. Although data span the 20^th^ century, over 99% of population change estimates are since 1980 and 73% since 1990. Species are also sampled in a skewed distribution with 60% of species appearing in just one or two locations (range  = 1 to 21; average 2.86, s.d. = 2.95; median  = 2, Appendix S1).

We classified species into three classes of growth form: annual herbs (902 observations, 97 species and subspecies); perennial herbs (662 observations; 121 species or subspecies); and woody shrubs and trees (118 observations; 35 species or sub-species). We also assigned species into one or more of 19 habitat types based on *Jepson Manual* species' habitat descriptions and predominant site type descriptions from the CNDDB records. Habitat types are general (e.g., deciduous woodland, grassland, sage scrub) for habitats that are dominant in the landscape, but more specific for edaphically extreme environments in which numerous rare species occur (e.g., vernal pools, alkali sinks, serpentine). Some species were lumped into a non-differentiated “other” category when habitat descriptions were vague (e.g., roadsides) or difficult to place into a particular habitat type (e.g., swales) These difficult to classify habitats are often indicative of stressful environments.

For each population, assessed plant population performance was estimated using an annualized lambda (λ_ann_) value following Akçakaya et al.[27];
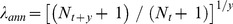
(1)where N_t_ is the first population estimate, N_t+y_ is the second population estimate, and y is the number of years between the estimates. Because individual locations may have population estimates from more than two years, each location may have multiple annualized λ estimates. A growing population is indicated by λ_ann_>1.0, while values less than one indicate a declining population. Owing to a strong skew (a small fraction of observations experience very large population change) in the resulting population growth measures, we transformed these measures by taking a natural log of the annual growth measure. We assessed λ_ann_ in three ways; using: i) all observed population changes (n = 1,682), ii) all individual populations (n = 795), and iii) the average of repeated estimates of population changes just for locations with multiple estimates (n = 341).

We categorized plant survey data by the specificity of the population estimate as follows: i) exact numbers less than 200; ii) exact numbers greater than 200 that likely represents a count or a close estimate (e.g., 1,347); or iii) round numbers that likely reflect an order of magnitude estimate (e.g., 1,000's). Exactness of a population estimate is likely strongly correlated with population size (e.g., small populations might be counted, large ones are estimated). Inexact estimates, however, can lead to spurious estimates of population change. For example, the difference between 5 and 10 individuals is likely to be exact and accurate while estimates of a large population may vary substantially among estimators. Hence, there is a trade-off between precision and bias in population data, so we tested for response differences in λ_ann_ depending on estimate specificity and initial population size.

Human density was assessed using census tracts as defined by the US Census Bureau statistics from the 2000 United States population census [28]. Census tracts are long-term county subdivisions containing between 2,500 and 8,800 people. We divided the census tract population by the area of each tract to get a human density (people/km^2^) for each plant location. Human density varied from zero (uninhabited islands of the coast) to 12,217 people/km^2^. Median density was 40.2 people/km^2^. Urban centers are often defined as having population densities of upwards of 400 people/km^2^ [29]. Our distribution of human densities was right-skewed, with 24% of observations from urban locations (>400 people/km^2^). We used the natural log of human density to partially normalize this distribution from which to predict performance and also used a non-parametric goodness-of-fit test, dividing the data into groups based on human density and λ_ann_.

We examined the relationship between λ and human density across the entire data set, by growth form (annual, perennial herbaceous plant, and woody plants), by habitat types (n = 19), and individually for the 25 species represented by 20 or more λ_ann_ estimates. We used the Human Threat Hypothesis (HTH) of Lawson et al. [23] as a basis for assessing whether λ_ann_ values decline with increasing human density. We expand beyond the work of Lawson et al [23] to test the the relationship between λ_ann_ and human density for specific growth forms (e.g., annual plants) and habitat types (e.g., wetlands). Our alternative hypothesis is that the variability in growth across growth form and habitat type to determine if some growth forms (e.g., annuals) exhibit more variability in population size. In general, greater variability in population size is expected in annuals than in perennial herbs or woody plants. In addition, we assessed whether mean growth rate is lower for particular growth forms or habitat types that are particularly prone to environmental degradation, (e.g., wetlands). This test serves to assess the sensitivity of the data to detect change. Finally, we combined the HTH with a null hypothesis of no difference among habitat types to test the hypothesis that relationship of λ_ann_ to human density does not differ among habitat types.

Urban populations change through time and densities estimated by the 2000 census may reflect impacts at the time of the CNDDB plant surveys with variable accuracy. Therefore, we tested for a relationship between λ_ann_ and human density using the data for the time period across which plant population change was estimated as a covariate. Our analysis consists of linear models of log transformed λ_ann_ as a function of growth form and habitat type, and log transformed human density and interactions among these variables. All analyses were done using JMP 9.0 (SAS institute).

Separately, we sought to understand conservation support across the gradient of high to low population density. We aggregated all recovery expenditures on behalf of 175 listed plant species in California using three years (2006–2008) of reported expenditures [30]–[32]. Eight species were not included as a consequence of recent listing action or taxonomic change. These 175 species represent over 95% of the total three-year endangered species expenditures. These expenditures are spent largely on managing populations and not on habitat acquisition, which is tracked separately. A separate treatment by Underwood et al. [33] reported on spending on behalf of acquisition, and is discussed in the context of our analysis. With a far smaller number of listed (n = 183) [34] rather than tracked rare species (n>2,000) [26], the geographic distribution of federally listed plant species (Fig. 1a) is less coastal than the distribution of California endemic plants [6].

We used CALJEP [35], a geospatial database of plant species distributions in California, to identify the distribution of these 175 federally listed species. We placed occurrences within a 1 km^2^ grid over the entire state. We counted polygons identified as “present” and “probably present” as occurrences, and “possible” and “not recorded” as absences [35]. We averaged the per 1 km^2^ expenditures across the three years based on an assumption that expenditures were evenly distributed across the range of a species. Most listed species are narrow endemics found in a very small portion of the state [4], [6], hence we find this an acceptable abstraction of the geographic distribution of spending on behalf of listed species. We summed the total expenditures within grid cells based on all listed species occurring in each grid cell when more than one species were found in a cell. We then mapped human density, based on census tract numbers, at the same spatial scale across the entire state, and estimated the relationship between expenditures and density at this 1 km^2^ grid cell scale for our coastal study region. In addition, we summed expenditures within census tracts and compared expenditures by human density at the census tract scale for the coastal counties for which we assessed λ_ann_ .

Finally, we also analyzed these data treating species as replicates. Here, we compared the average human density in all tracts in which a species occurs to the total three years of expenditures on behalf of the species. Results were similar using either species or census tract as the unit of observation. We focused on the data that depict spending by census tract as it relates more directly to our geographic depiction of the distribution of spending. All variables were log transformed to better fit the expectation of normality.

## Results

### Plant population growth by human density

**Figure 2:**
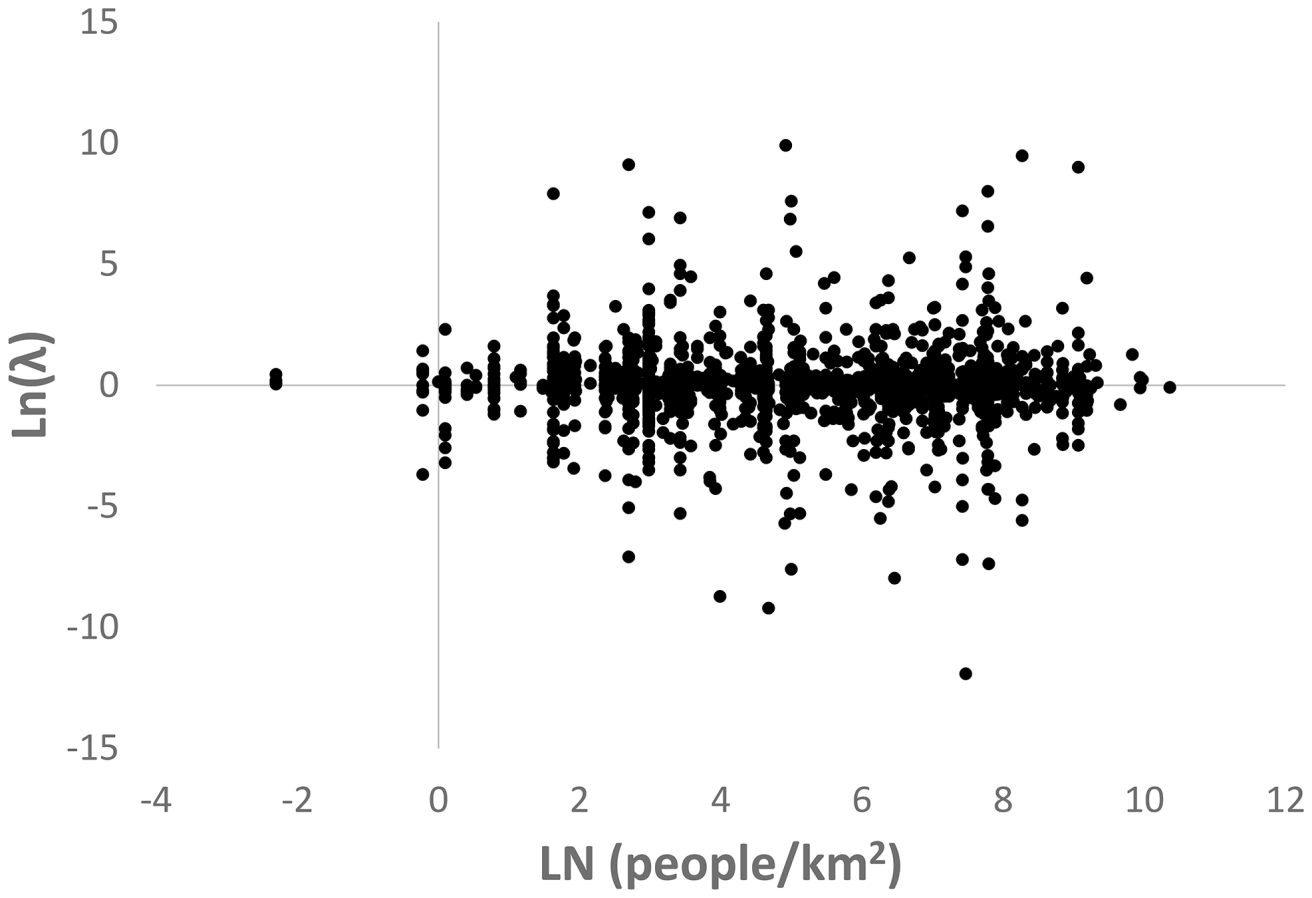
A scatterplot of the relationship between the natural log of annualized plant population growth (λ_ann_) and human density (people/km^2^). The plot shows no relationship between human density and λ_ann_.

Our results agree with those of Lawson et al. [17] in finding no relationship between human population density and λ_ann_ (n = 1,682, F = 0.038, p = 0.85, coefficient  = −0.003) (Table 1, Fig. 2). This strongly non-significant result persists irrespective of how we assessed the data including across parametric versus nonparametric tests and all subsets of data based on sampling, sampling date, or sample specificity (Table 1). We found no bias in the examination of residuals for any of these tests. The effect size of human density on λ_ann_, as estimated by 1,000 replicates of a randomized bootstrap sample of 1,000 observations, was nearly zero (mean correlation coefficient  = −0.006; s.d. = 0.0235).

**Table 1:** Plant population growth response as predicted by human density using 13 different tests of the relationship performed to assure consistency of results depending on how we treated (A) repeat sampling within a plant population; (B) violations of normal distributions driving non-parametric considerations; (C) initial population size and the ease of gaining an accurate population assessment; (D) when the plant population was assessed; and (E) growth form.

	Criteria	N	P	Coefficient
A. Unit of observations				
	All population change estimates	1682	0.845	−0.0032
	Average of observations from each population	795	0.519	−0.0083
	Average of observations with multiple estimates	341	0.596	−0.008
B. Nonparametric correlation*				
	All population change estimates, Kendall's tau	1682	0.4	−0.0014
	All population change estimates, Spearmans p	1682	0.406	−0.0203
C. Population estimate specificity				
	Population size less than 200	654	0.613	−0.011
	Exact estimate, population larger than 200	504	0.51	0.232
	Rounded number estimate, population >200	521	0/296	−0.032
D. Population count end date				
	Population change end date prior to 1990	446	0.221	−0.0435
	Population change end date 1990 or later	1234	0.527	0.0116
E. Life Form				
	Annual herbaceous plants	902	0.814	0.0067
	Perennial herbaceous plants	660	0.857	0.0029
	Woody trees and shrubs	118	**0.046**	−0.0498

*-nonparametric versions of most of correlations by population for varying units of observations (A) and estimate specificity (B) were similarly, not significant and had correlations close to 0.

We observed no effect of year on λ_ann_ (n = 1,682; F = 0.42; p = 0.84). There was a slight positive relationship between initial plant population size and human density (n = 1,682; F = 10.3; p = 0.001; coefficient  = 0.095) and a negative correlation between λ_ann_ and initial population size (n = 1,682; F = 141.7; p<0.001; coefficient  = −0.154). This combination of results should make it more likely to find an adverse impact of human population on λ_ann_, yet we do not find this to be the case.

We also conducted a goodness-of-fit test on data classified into categories based on human density and λ_ann_. Human density classes consisted of rural (<40 people/km^2^, n = 785), peri-urban (40–400 people/km^2^, n = 488), and urban (>400 people/km^2^, n = 409). We classified λ_ann_ into shrinking (ln(λ_ann_)< −0.2; n = 560), stable (−0.2<ln(λ_ann_)<0.2, n = 595) and growing (ln(λ_ann_)>0.2, n = 520) populations approximately by equal frequency categories. Goodness-of-fit test results were significant (chi square  = 10.79; df = 4; p = 0.029; Table 2). The pattern of this significance suggests an increase in variability of plant growth responses in urban environments. We found more observations of both λ_ann_ growth and retraction in urban environments and fewer than expected stable transitions (−0.2>λ_ann_>0.2). Conversely, the lowest human density sites exhibited fewer population declines and more stable transitions than expected (Table 2). We further examined this relation bycreating a more stringent criteria for growing and shrinking populations, where values between −1 and +1 were considered stable, and larger values were characterized as collapse (ln(λ_ann_)< −1.0; n = 218) or growth (ln(λ_ann_)>1; n = 226). Although the general pattern observed above remained, the test result was not significant (chi-square  = 4.46; p = 0.35)

**Table 2:** A contingency table of λ_ann_ relative to human density showing observed numbers of populations within each class and the expected numbers parenthetically; goodness-of-fit likelihood ratio  = 10.8 (p = 0.03).

	Plant population change		
Human Population	Shrink	Stable	Grow
0–40 people/km^2^	245 (261.4)	298 (277.7)	242 246.0)
40–400 people/km^2^	110 (109.2)	125 (116.0)	93 (102.8)
>400 people/km^2^	205 (189.4)	172 (201.3)	192 (178.3)

Given that the chi-square tests suggest that urban populations may be more variable than rural ones, and that variability can lead to decreased persistence likelihoods, we further assessed variability in λ_ann_. We restricted our assessment to observations with at least five repeat population change estimates (six survey dates). We found no relationship between the coefficient of variation (standard deviation/mean) as a function of human density (n = 80; F = 0.43; p = 0.51). An alternative explanation for increased variability of λ_ann_ in urban environments could be a differential distribution of life forms, with annual herbaceous plants found, on average, in higher human densities. This, in fact, is the case (one-way ANOVA, n = 1,682, df = 2, F = 25.7; p<0.001). Half (50.4%) of all observations were annual plants, yet 58.7% of urban λ_ann_ were from annual plants. These effects, however, are small. A linear model predicting λ_ann_ by growth form and human density yields no significant effects or interactions.

Assessing λ_ann_ by growth form indicated no differences among life forms in their population responses to human density for annual or perennial herbaceous plants (Table 1). Woody trees and shrubs, however, exhibited a significant decrease in λ_ann_ with increasing human density (Table1). This is the single significant result we observed relating λ_ann_ to human density. Examining the distribution of life form across human density showed a significant difference (ANOVA; n = 1,682, F = 25.70; p<0.001) with woody plants being found at significantly lower human densities.

Finally, we assessed λ_ann_ by species and by habitat type. We found no significant (p<0.1) patterns with human density, and observed an even balance of those with positive (n = 11) and negative (n = 11) coefficients relative to human density for the 22 species with 20 or more λ_ann_ estimates (Table 3). We constructed independent tests of λ_ann_ versus human density for 19 habitat types. Again, we found no significant (p = 0.1) correlations in either direction (Table 4). Further, we achieved the same results when we reduced the number of data points by removing data of lower overall quality and restricting estimates to those data with specific estimates of the number of plants (Table 4). We observed no effect of human density, nor an interaction of habitat type by population. We did not correct for family error rates in either case as not a single test was significant even at a 0.10 level. There were considerable differences in the distribution of habitat types with respect to human density, but no relationship between the distribution of human densities sampled within a habitat type and the overall mean performance for that habitat type (Table 4).

**Table 3:** Correlations of λ_ann_ with human density for the 22 species with 20 or more individual observations.

Species	n	F	P	Coefficient
*Acanthomintha ilicifolia*	38	0.37	0.55	0.074
*Amsinckia grandiflora*	21	0.848	0.37	−0.167
*Astragalus brauntonii*	23	1.003	0.33	0.142
*Blennosperma bakeri*	27	0.027	0.87	−0.039
*Camissonia benitensis*	35	0.001	0.97	0.009
*Clarkia franciscana*	66	0.89	0.35	0.347
*Cordylanthus maritimus* ssp. *maritimus*	20	0.045	0.84	−0.063
*Cordylanthus maritimus* ssp. *palustris*	57	2.71	0.11	−0.101
*Cordylanthus mollis* ssp. *mollis*	26	0.032	0.86	0.045
*Dichanthelium lanuginosum* var. *thermal*	30	0.182	0.67	−0.049
*Dudleya multicaulis*	25	0.03	0.86	0.01
*Dudleya setchellii*	11	1.82	0.4	0.063
*Fritillaria liliacea*	54	0.126	0.72	−0.02
*Gilia tenuiflora* ssp. *arenaria*	22	0.297	0.59	−0.103
*Helianthella castanea*	20	1.77	0.2	0.219
*Hesperolinon congestum*	26	0.013	0.91	0.012
*Holocarpha macradenia*	101	0.552	0.46	0.083
*Lasthenia conjugens*	24	0.1	0.76	−0.184
*Lupinus tidestromii*	21	0.047	0.83	−0.055
*Pentachaeta lyonii*	34	0.566	0.46	0.083
*Phacelia insularis* var. *continentis*	26	0.001	0.97	−0.009
*Triphysaria floribunda*	35	0.301	0.59	−0.049

No significant (p<0.1) correlations and equal numbers of positive and negative correlation coefficients were observed.

**Table 4:** Summary statistics for correlation of population performance (natural log of annualized mean λ) with human density.

	Population Growth	All Observations			Highly specific estimates			Distribution of occurrences by human population density		
Habitat	Mean	n	coefficient	p	n	coefficient	p	Median	F	p
	ln(ë)							People/km2		
**Conifer Forest**	0.272	60	−0.014	0.81	49	−0.087	0.25	14.1	6.57	**0.011**
**Alkali Sink**	0.191	35	−0.08	0.68	18	−0.116	0.84	13.8	3.32	0.069
**Desert**	0.179	29	−0.008	0.9	22	−0.009	0.92	5.7	13.3	**0.003**
**Deciduous Woodland**	0.111	10	−0.001	0.97	8	0.026	0.63	35.3	0.902	0.342
**Vernal Pool**	0.103	162	0.04	0.64	54	−0.013	0.21	61	1.51	0.219
**Sand Dunes**	0.075	194	0.003	0.94	162	0.012	0.78	40	0.316	0.574
**Freshwater Wetland**	0.074	208	−0.024	0.56	147	−0.009	0.86	7.8	26.23	**0.0001**
**Ocean Bluffs**	0.069	64	−0.021	0.66	54	0	0.99	6.2	19.51	**0.0001**
**Serpentine**	0.04	300	−0.01	0.76	249	0.004	0.83	59	9.09	**0.003**
**Sagescrub**	0.012	249	0.032	0.48	215	0.034	0.47	38.6	7.04	**0.008**
**Chaparral**	0.011	288	0.016	0.68	227	0.046	0.92	59.3	2.04	0.154
**Woodland**	−0.015	161	−0.037	0.3	114	−0.026	0.5	20.4	34.63	**0.0001**
**Riparian**	−0.02	48	−0.03	0.57	16	0.052	0.56	7.8	2.69	0.101
**Grassland**	−0.03	752	0.005	0.83	511	0.033	0.26	59.3	6.71	**0.01**
**Rocky Slopes**	−0.035	104	0.007	0.78	77	0.017	0.57	40	0.351	0.553
**Other (stress)**	−0.104	60	0.047	0.31	51	0.047	0.4	7.4	0.455	0.5
**SaltMarsh**	−0.184	154	−0.094	0.16	91	−0.138	0.14	7.2	3.31	0.07

Each regression was conducted on two subsets of data: all observations; and data that meet our data quality criteria of having high precision. The key point to note is that there are no significant relationships. Also of note are the differences in mean λ_ann_ by habitat. Habitats are ranked from the highest mean λ_ann_ to the lowest (left-most data column). Many individual habitats were found in significantly more urban or rural environments (right columns), but the overall habitat performance was not predicted by either more urban or rural distributions. Values <0.05 are in bold face.

In summary, we found no support for the hypothesis that increasing human density results in decreasing plant performance other than for the case of woody plants. Among the 19 habitat types assessed, mean λ_ann_ varied with 12 habitat types exhibiting a positive net growth rate and seven habitat types with an average negative growth rate. No individual habitat type showed significant variation in λ_ann_ across a gradient of human density within habitats (Table 4).

### Plant Endangered Species Expenditures by Human Population Density

Our assessment of endangered species expenditures shows that there is a weak, but positive, relationship between the natural log of human density and the natural log of endangered species expenditures using both census tract (coefficient  = 0.186; r^2^ = 0.029; p<0.001 n = 5,064) and species (coefficient  = 0.112; r^2^ = 0.025; p<0.039; n = 171) as the replicate. We tried several additional transformations but the results did not substantively vary by transformation. Investigating the effect of range size on expenditures suggest that species with larger distributions have slightly more expenditures and that controlling for this effect reverses the effect of human density on expenditures (coefficient  = −0.17; r^2^ = 0.037; p<0.01 n = 171).

This coarse evaluation masks important detail, as San Diego County receives far more endangered species recovery expenditures than any other county in our study region (or likely, in the US) [26]–[28] (Table 5, Fig. 1). This county contains both regions of high and low human density. Excluding San Diego County actually increases both the coefficient and the fit of the positive relationship between human density and spending among census tracts (coefficient  = 0.234; r^2^ = 0.081; p<0.001 n = 4,459).

**Table 5:** County level statistics of human population for coastal California from the San Francisco Bay metropolitan region southward and spending on behalf of federally listed endangered and threatened plant species from 2006–2008.

COUNTY	POPULATION	Mean $/km^2^	Coefficient	P
Alameda	1,443,741	57.47	−0.003	0.04
Contra Costa	948,816	58.87	0.008	0.0003
Los Angeles	9,453,140	85.69	0.002	<0.0001
Marin	246,104	90	−0.001	0.518
Monterey	407,907	372.35	0.035	0.0001
Napa	119,901	8.36	−0.003	0.133
Orange	2,852,710	64.61	−0.003	0.0019
San Benito	40,838	4.8	−0.003	0.157
San Diego	3,056,509	1591.99	0.012	0.0003
San Francisco	790,240	128.24	0.001	0.0004
San Luis Obispo	207,490	108.03	0.022	0.18
San Mateo	708,709	131.97	0.037	0.013
Santa Barbara	495,933	16.49	−0.001	0.435
Santa Clara	1,675,965	19.66	−0.003	<0.001
Santa Cruz	250,245	80.95	0.004	0.008
Solano	394,542	67.99	0.003	0.565
Sonoma	458,614	51.02	0.012	<0.0001
Ventura	806,420	50.99	0.002	0.412

Human density (people/km^2^) estimates are from the 2000 US census, summarized in the California Department of Finance (www.dof.ca.gov). Expenditure estimates are from the US Fish and Wildlife endangered and threatened species expenditure reports for 2006 through 2008. Expenditures are reported by species and we used species distribution maps to identify species within counties and averaged species expenditures within census tracts across distributions. Regression coefficients and p-values are for human density versus the expenditures by census tract.

Among the 17 counties we included in this study, the relationship between human density and spending was significant in 10 counties, of which seven (Contra Costa, Los Angeles, Monterey, San Diego, San Francisco, Santa Cruz, Sonoma) were positive and three (Alameda, Orange, Santa Clara) were negative (Table 5). At a coarser scale, counties with very high average human densities tended to be on the highest end of expenditures (San Diego, Los Angeles Counties) or toward the low end of expenditures (Orange, Santa Clara Counties) with no apparent relationship between spending and human density.

## Discussion

**Figure 3:**
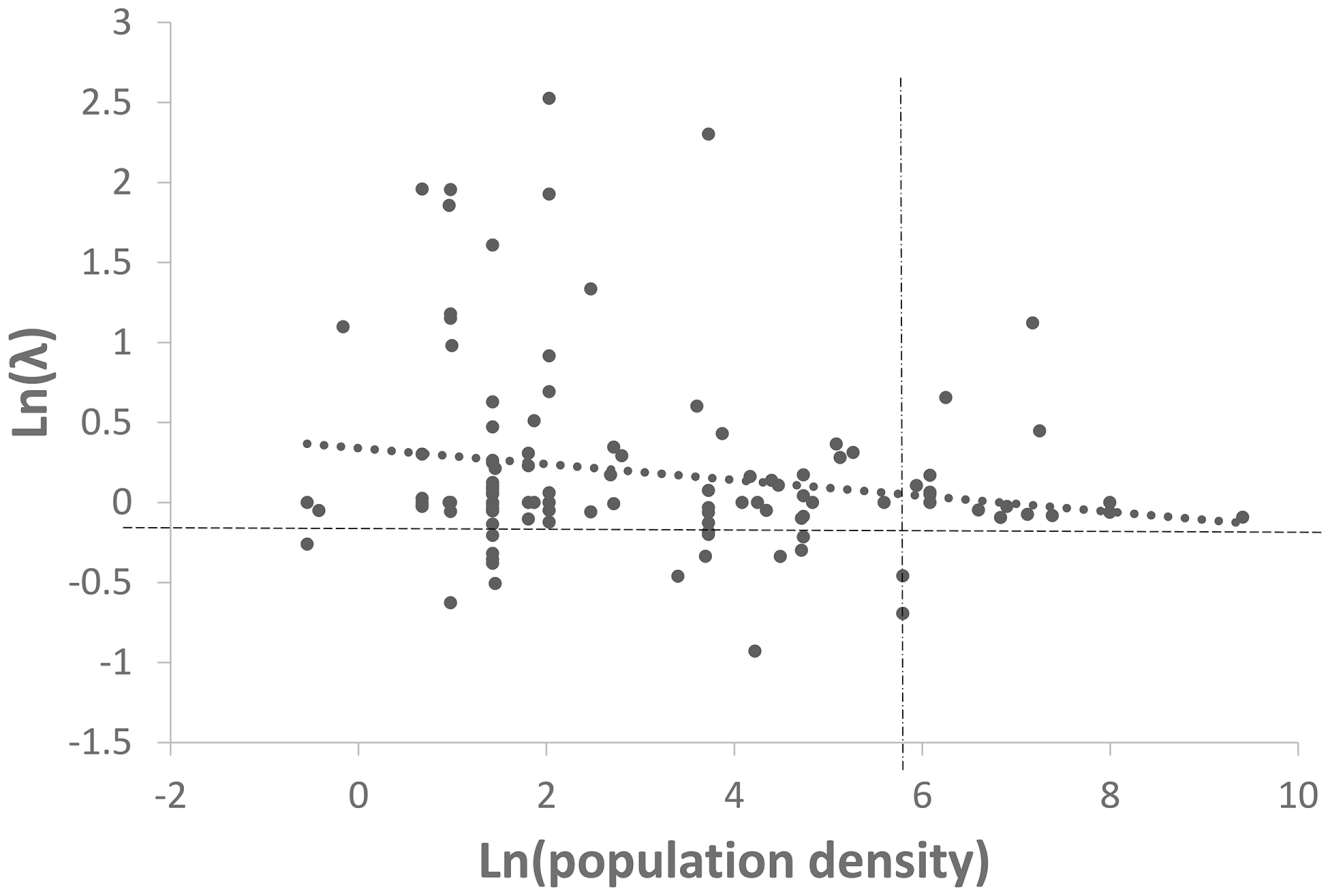
The correlation between human population density and plant population growth (λ) for woody plants. The only significant correlation observed between human density and plant performance (λ_ann_) was for woody plants (n = 118, F = 4.06; p = 0.046; coefficient  = −0.050). However this relationship is driven by high λ_ann_ in rural areas as opposed to strong negative λ_ann_ in urban settings. The horizontal dashed line represents no population change, the vertical dashed line separates urban (right) from non-urban (left) populations, with the dotted line representing the best fit correlation.

Our results both support and strengthen the conclusions of Lawson et al. [23] that plant populations perform equally well across the gradient from rural to urban locations. Using a larger dataset over a more extensive coastal region, and numerous additional analyses, we find no evidence that at-risk plant populations perform poorly in areas with high human density. Out of 65 tests of λ versus human population density, only a single result was significant. That result suggests that woody plants have lower average growth rates in areas of high human density than they do in more rural areas. However, this result is brought into question by the observation that only 16 of our 118 observations were from urban (>400 people/km) locations, and these represented just 6 of 35 woody species. Among the four woody species found in both urban (>400 people/km^2^) and non-urban (<400 people/km^2^) areas, three actually had higher average growth rates in urban populations than in their rural ones. In addition, this negative correlation is not driven by low λ_ann_ values in urban populations, but high λ_ann_ values in rural populations (Fig. 3).

The results of our study strongly suggest a potential for successful plant conservation efforts within the urban environments of California. We do not mean to imply, however, that populations of at-risk plants are not threatened by urban environments. Habitat loss, and the extirpation of populations, remains a critical issue. Our data suggest that protecting these populations from habitat loss may provide opportunities for protection of these at-risk biological resources. By focusing on those populations that remained extant across survey periods, we found that λ_ann_ was no different across the spectrum from rural to urban environments.

A goal of plant conservation should be to protect viable populations over long time periods. The CNDDB data do not provide the capacity to conduct viability assessments using these data. Our observation of slightly greater rates of inter-annual variability for plants in areas of high human density, and high inter-annual variability generally, may result in higher long-term vulnerability of urban populations. Analyzing magnitude of this effect is beyond the scope of this project and the capacity of these data.

Our results expand on prior results. We consider variability in response in a variety of attributes, including habitat, growth form and time period, analyzing the CNDDB data in far more detail than previous studies. Second, we distinguish among growth forms and habitat types to assess performance across the gradient from rural to urban habitats. Our results suggest that specialized habitat types, such as alkali sinks, vernal pools and desert, may be more resilient to threats associated with urbanization as demonstrated by larger average λ values than other habitat types (Table 4). If homogenization of urban floras is driven by invasive species [15], [16], then it stands to reason that habitat types that are less prone to invasion may be more resilient in urban systems. This does not help explain the relatively large net positive growth rates among species found in coniferous or deciduous forests, but may help explain the generally negative mean growth rates for riparian, grassland and salt marsh species (Table 4).

An alternative possible interpretation of the Lawson et al. [23] and these results is that the CNDDB data are not sufficiently specific and detailed to assess differences in λ_ann_. The CNDDB data are haphazardly collected by a broad and diverse suite of individuals across a long timeframe using different methods. Our finding—that habitat types differ in performance in ways that we would predict based on habitat resilience—is encouraging because it supports the assertion that these data provide a signal of performance that can be assessed. If there were no habitat type differences, then we might conclude that the data are simply too coarse to distinguish performance. However, since λ_ann_ values differ across habitat types, and those habitat types that are less prone to degradation through invasive species appear more resilient, this provides evidence that our data are, in fact, informative.

Finding no relationship between λ_ann_ and human density, we reinforce the assertion by Lawson et al. [23] that plant performance within protected habitats is not diminished simply by virtue of having a high local human density. This is encouraging given the significant conservation investment in and around major California urban areas [33]. Our study was not designed to determine why spending is allocated differentially across urban areas, with San Diego County receiving far more recovery funding than other California urban centers of high rare plant density. Nevertheless, we find it likely that some of the differential spending in California is driven by systematic conservation planning through tools such as the state's Natural Community Conservation Planning (NCCP) [36] and the Federal Habitat Conservation Planning (HCP) [37] .

Our geographic treatment of endangered species recovery expenditures is, necessarily, an abstraction of real expenditures. We do not know exactly where money was applied or to which species. However, our intent is to present an overview of the geography of federal expenditures in support of plant conservation, and not to assess the effectiveness of those reported expenditures.

Examining this result geographically, there is a complex relationship between human density (Fig. 1c), listed species density (Fig. 1a), and expenditures (Fig. 1b). Rural and medium density regions appear to uniformly receive little support for their endangered species (Fig. 1b, 1c). Similarly, the San Francisco and Los Angeles metropolitan regions have high human densities, high rare species richness, and modest expenditures (Fig. 1). In contrast, the San Diego region, with high human density, is rich in listed species and these species garner high levels of financial support (Fig. 1). This region appears unique in that respect. We speculate that the plants of San Diego County garner more funding because of the extensive multi-species conservation plans in place within the region [38].

The US Endangered Species expenditure data suggest that there are significant resources being applied to urban conservation in San Diego County, but not generally across the region. Underwood et al. [33], analyzing land protection expenditures in California, also found a large focus on spending along the urbanized coast of California. Schwartz et al. [4] made the case that providing for protection in urban environments can have collateral positive impacts on conservation through social engagement in the process of conservation. Whether by chance or design, evidence that λ_ann_ does not diminish with human density provides an endorsement of conservation expenditures in California. This is simply because such a large percentage of the unique flora of California is isolated to coastal, and often urban, regions of the state.

If plants are generally in need of protection, and human dominated landscapes are both rich in populations of rare species and under the most severe immediate threat, then it is sensible to skew investment toward these urban areas (San Francisco, Los Angeles, San Diego). However, this would be a poor investment of conservation resources if there were evidence that protected populations in urban environments were less likely to persist. Our study helps to assure conservation managers that these populations, once protected, do not appear to be at undue risk simply by virtue of urban proximity.

The case for investing in urban plant conservation [4] is further strengthened by examining extinction processes in urban environments [11], [14], [39]. The California Native Plant Society (CNPS) maintains a database on rare and endangered plants. This list includes 27 species considered extirpated in California [26]. Among these 27 extirpated species, 12 were formerly found within our study region. Among these 12 regional extirpated species, only two (*Ribes divericatum* var *parishii*, 1980, Los Angeles and San Bernardino counties; *Castilleja uliginosa*, 1987, Sonoma county) have been observed since 1954. In other words, just two of 12 documented extirpations have occurred since the onset of modern conservation measures such as the Endangered Species Act. These data do not indicate how many populations of plants may have been lost through time, nor do they report on individual county extirpations, but they do give an overall indication that California, with a high number of narrow endemics (defined as occurring in 1–2 counties [23]), has lost very few species through 30 years of conservation management. Given these arguments, we maintain that conservation investment in California urban centers can successfully protect rare plants over the medium term and provide strong incentives for local conservation value. Given the parallel conservation need to engage the urban population in protecting nature [21], investment of limited resources in urban plant conservation appears as likely as any to succeed.

## Supporting Information

Appendix S1 **A list of all populations found in the California Natural Diversity Database assessed for the 17 county region of coastal California from Main County to San Diego County.**
(DOCX)
