# Supplementary material for: Conservation Investment for Rare Plants in Urban Environments
Source: PLoS One. 2013 Dec 31;8(12):e83809. doi: 10.1371/journal.pone.0083809 (PMC3877106; doi:10.1371/journal.pone.0083809)
Supplement: Appendix S1 — A list of all populations found in the California Natural Diversity Database assessed for the 17 county region of coastal California from Main County to San Diego County. (DOCX) [file pone.0083809.s001.docx]

**Appendix S1.** A list of all populations found in the California Natural Diversity Database assessed for the 17 county region of coastal California from Main County to San Diego County.

*Abies bracteata*

*Abronia umbellata ssp. breviflora*

*Acanthomintha duttonii*

*Acanthomintha ilicifolia*

*Adolphia californica*

*Agrostis blasdalei*

*Agrostis clivicola var. punta-reyesensis*

*Allium hickmanii*

*Allium howellii var. clokeyi*

*Allium sharsmithiae*

*Alopecurus aequalis var. sonomensis*

*Ambrosia pumila*

*Amorpha californica var. napensis*

*Amsinckia grandiflora*

*Amsinckia lunaris*

*Arabis hoffmannii*

*Arctostaphylos auriculata*

*Arctostaphylos cruzensis*

*Arctostaphylos densiflora*

*Arctostaphylos glandulosa ssp. crassifolia*

*Arctostaphylos hookeri ssp. hearstiorum*

*Arctostaphylos hookeri ssp. hookeri*

*Arctostaphylos hookeri ssp. ravenii*

*Arctostaphylos otayensis*

*Arctostaphylos pallida*

*Arctostaphylos rainbowensis*

*Arctostaphylos stanfordiana ssp. decumbens*

*Arctostaphylos virgata*

*Arenaria paludicola*

*Astragalus brauntonii*

*Astragalus claranus*

*Astragalus douglasii var. perstrictus*

*Astragalus nevinii*

*Astragalus oocarpus*

*Astragalus pycnostachyus var. pycnostachyus*

*Astragalus tener var. tener*

*Astragalus traskiae*

*Atriplex depressa*

*Atriplex joaquiniana*

*Atriplex pacifica*

*Atriplex vallicola*

*Baccharis malibuensis*

*Balsamorhiza macrolepis var. macrolepis*

*Berberis nevinii*

*Berberis pinnata ssp. insularis*

*Blennosperma bakeri*

*Blennosperma nanum var. robustum*

*Blepharizonia plumosa*

*Bloomeria clevelandii*

*Brodiaea filifolia*

*Brodiaea orcuttii*

*Calamagrostis crassiglumis*

*California macrophylla*

*Callitropsis abramsiana*

*Calochortus clavatus var. recurvifolius*

*Calochortus dunnii*

*Calochortus obispoensis*

*Calochortus palmeri var. palmeri*

*Calochortus plummerae*

*Calochortus pulchellus*

*Calochortus striatus*

*Calochortus weedii var. intermedius*

*Calycadenia villosa*

*Calystegia subacaulis ssp. episcopalis*

*Camissonia benitensis*

*Camissonia hardhamiae*

*Campanula californica*

*Carex obispoensis*

*Castilleja affinis ssp. neglecta*

*Castilleja ambigua ssp. humboldtiensis*

*Castilleja grisea*

*Caulanthus californicus*

*Caulanthus coulteri var. lemmonii*

*Ceanothus gloriosus var. porrectus*

*Ceanothus hearstiorum*

*Ceanothus maritimus*

*Ceanothus masonii*

*Centromadia parryi ssp. australis*

*Centromadia parryi ssp. congdonii*

*Cercocarpus traskiae*

*Chlorogalum purpureum var. purpureum*

*Chlorogalum purpureum var. reductum*

*Chorizanthe blakleyi*

*Chorizanthe breweri*

*Chorizanthe cuspidata var. villosa*

*Chorizanthe orcuttiana*

*Chorizanthe parryi var. fernandina*

*Chorizanthe pungens var. pungens*

*Chorizanthe rectispina*

*Chorizanthe robusta var. robusta*

*Chorizanthe valida*

*Cirsium andrewsii*

*Cirsium fontinale var. campylon*

*Cirsium fontinale var. fontinale*

*Cirsium fontinale var. obispoense*

*Cirsium hydrophilum var. vaseyi*

*Cirsium loncholepis*

*Cirsium occidentale var. compactum*

*Cirsium rhothophilum*

*Clarkia concinna ssp. raichei*

*Clarkia franciscana*

*Clarkia speciosa ssp. immaculata*

*Cordylanthus maritimus ssp. maritimus*

*Cordylanthus maritimus ssp. palustris*

*Cordylanthus mollis ssp. hispidus*

*Cordylanthus mollis ssp. mollis*

*Cordylanthus palmatus*

*Cordylanthus rigidus ssp. littoralis*

*Corethrogyne filaginifolia var. linifolia*

*Cryptantha ganderi*

*Cymopterus deserticola*

*Deinandra conjugens*

*Deinandra increscens ssp. villosa*

*Deinandra minthornii*

*Delphinium bakeri*

*Delphinium hesperium ssp. cuyamacae*

*Delphinium hutchinsoniae*

*Delphinium luteum*

*Delphinium recurvatum*

*Delphinium variegatum ssp. kinkiense*

*Delphinium variegatum ssp. thornei*

*Dichanthelium lanuginosum var. thermale*

*Dieteria asteroides var. lagunensis*

*Dirca occidentalis*

*Dithyrea maritima*

*Dodecahema leptoceras*

*Downingia pusilla*

*Dudleya abramsii ssp. bettinae*

*Dudleya blochmaniae ssp. blochmaniae*

*Dudleya brevifolia*

*Dudleya candelabrum*

*Dudleya cymosa ssp. ovatifolia*

*Dudleya densiflora*

*Dudleya multicaulis*

*Dudleya parva*

*Dudleya setchellii*

*Dudleya stolonifera*

*Dudleya traskiae*

*Eriastrum luteum*

*Erigeron supplex*

*Eriogonum butterworthianum*

*Eriogonum giganteum var. compactum*

*Eriogonum giganteum var. formosum*

*Eriogonum nervulosum*

*Eriogonum nortonii*

*Eriophyllum lanatum var. hallii*

*Eryngium aristulatum var. parishii*

*Erysimum capitatum var. angustatum*

*Erysimum menziesii ssp. menziesii*

*Erysimum teretifolium*

*Eschscholzia rhombipetala*

*Ferocactus viridescens*

*Fritillaria falcata*

*Fritillaria lanceolata var. tristulis*

*Fritillaria liliacea*

*Fritillaria ojaiensis*

*Fritillaria viridea*

*Galvezia speciosa*

*Gilia capitata ssp. chamissonis*

*Gilia tenuiflora ssp. arenaria*

*Gratiola heterosepala*

*Grindelia hirsutula var. hallii*

*Grindelia hirsutula var. maritima*

*Helianthella castanea*

*Hesperolinon breweri*

*Hesperolinon congestum*

*Hesperolinon sp. nov. "serpentinum"*

*Hibiscus lasiocarpus*

*Hoita strobilina*

*Holocarpha macradenia*

*Horkelia marinensis*

*Horkelia tenuiloba*

*Ipomopsis tenuifolia*

*Isocoma arguta*

*Lasthenia burkei*

*Lasthenia conjugens*

*Lavatera assurgentiflora ssp. glabra*

*Layia carnosa*

*Layia discoidea*

*Layia heterotricha*

*Lepechinia cardiophylla*

*Lepidium flavum var. felipense*

*Leptosiphon croceus*

*Lessingia germanorum*

*Lilium maritimum*

*Limnanthes douglasii ssp. sulphurea*

*Limnanthes gracilis ssp. parishii*

*Limnanthes vinculans*

*Linanthus orcuttii*

*Lithophragma maximum*

*Lotus argophyllus var. adsurgens*

*Lotus dendroideus var. traskiae*

*Lotus nuttallianus*

*Lupinus guadalupensis*

*Lupinus ludovicianus*

*Lupinus nipomensis*

*Lupinus tidestromii*

*Malacothamnus fasciculatus var. nesioticus*

*Malacothamnus hallii*

*Malacothamnus palmeri var. involucratus*

*Malacothrix indecora*

*Malacothrix saxatilis var. arachnoidea*

*Mimulus fremontii var. vandenbergensis*

*Monardella frutescens*

*Monardella hypoleuca ssp. lanata*

*Monardella macrantha ssp. hallii*

*Monardella stoneana*

*Monardella viminea*

*Nasturtium gambelii*

*Navarretia leucocephala ssp. bakeri*

*Navarretia leucocephala ssp. pauciflora*

*Navarretia nigelliformis ssp. radians*

*Navarretia prostrata*

*Neostapfia colusana*

*Nolina interrata*

*Oenothera deltoides ssp. howellii*

*Orcuttia californica*

*Orobanche valida ssp. valida*

*Packera ganderi*

*Pedicularis dudleyi*

*Penstemon newberryi var. sonomensis*

*Pentachaeta lyonii*

*Phacelia insularis var. continentis*

*Phacelia stellaris*

*Piperia yadonii*

*Plagiobothrys diffusus*

*Pleuropogon hooverianus*

*Poa atropurpurea*

*Pogogyne clareana*

*Pogogyne nudiuscula*

*Polygonum marinense*

*Potentilla hickmanii*

*Sanicula maritima*

*Sanicula saxatilis*

*Scrophularia atrata*

*Sidalcea calycosa ssp. rhizomata*

*Sidalcea hickmanii ssp. parishii*

*Silene verecunda ssp. verecunda*

*Streptanthus albidus ssp. albidus*

*Streptanthus albidus ssp. peramoenus*

*Streptanthus brachiatus ssp. brachiatus*

*Streptanthus brachiatus ssp. hoffmanii*

*Streptanthus hispidus*

*Streptanthus morrisonii*

*Streptanthus niger*

*Suaeda californica*

*Symphyotrichum lentum*

*Thermopsis californica var. semota*

*Trifolium depauperatum var. hydrophilum*

*Trifolium polyodon*

*Trifolium trichocalyx*

*Triphysaria floribunda*

*Tuctoria mucronata*
